# Evidence-Based Patient Classification for Traditional Chinese Medicine

**DOI:** 10.1155/2015/168343

**Published:** 2015-07-21

**Authors:** Guo-Zheng Li, Ka-Fai Chung, Josiah Poon

**Affiliations:** ^1^Data Center of Traditional Chinese Medicine, China Academy of Chinese Medical Science, Beijing 100700, China; ^2^Department of Psychiatry, The University of Hong Kong, Hong Kong; ^3^School of Information Technologies, The University of Sydney, Sydney, NSW 2006, Australia

Some form of categorization or subdivision of disease states is inevitable in all branches of medicine. According to the traditional Chinese medicine (TCM) theory, the patterns of bodily disharmony are described in terms of eight major parameters:* yin* and* yang*,* external* and* internal*,* hot* and* cold*, and* excess* and* deficiency*. Additional systems such as* qi*,* blood*, and* body-fluid* differentiation and* zang fu* (organ) differentiation are also used. To enhance wider acceptance, these TCM categories have to have sufficient reliability and validity. There are interrater and test-retest reliability and four major types of validity: concurrent, predictive, construct, and content. As the TCM theory originated from ancient medical texts and the classification is based on symptoms and signs, the key shortcomings are the highly subjective diagnostic process and the lack of scientific support for the TCM classification. In this special issue, the evidence base of the TCM classification was investigated.

In the paper “Advances in Patient Classification for Traditional Chinese Medicine: A Machine Learning Perspective,” the authors introduced the machine learning algorithms for sign classification, syndrome differentiation, and disease classification. Clinical features, as derived from inspection, auscultation, olfaction, and palpation, and patients' dataset from medical records could be analyzed by* k* nearest neighbor, support vector machine, linear discriminant analysis, naive bayes, decision tree, artificial neural network, graphical models, multilabel learning, deep learning, or clustering analysis. The use of machine learning algorithms is a step toward enhancing data reliability, but the accuracy, as compared to experienced TCM practitioners, is still suboptimal. We believe that the machine learning algorithms are best used to deal with large and complex dataset, while the application in sign classification merits further investigation.

With the help of computer technology, the paper “Significant Geometry Features in Tongue Image Analysis” categorized five different tongue shapes: rectangular, acute triangle, obtuse triangle, circle, and square. The authors found that tongue shape can be used to distinguish healthy and disease states, with an average accuracy of 76%. Further testing of the role of tongue shape in TCM classification is needed.

Two papers introduced novel statistical approaches to assist TCM classification. In the paper “Mining Symptom-Herb Patterns from Patient Records Using Tripartite Graph,” the authors designed a data mining approach to examine the relationship between symptom, syndrome, and herb. This tripartite information network derives more accurate information than linking symptom and herb alone. In the paper “A Novel Classification Method for Syndrome Differentiation of Patients with AIDS,” the authors found that a novel machine learning algorithm, minimum reference set-based multiple instance learning, was superior to other machine learning algorithms for TCM classification.

A few papers examined the clinical application of TCM classification. In the paper “Yang Deficiency Body Constitution Acts as a Predictor of Diabetic Retinopathy in Patients with Type 2 Diabetes: Taichung Diabetic Body Constitution Study,” 673 patients with diabetes were examined using a body constitution questionnaire. The authors showed that* yang deficiency* was an independent predictor of a lower risk of diabetic retinopathy, suggesting that TCM classification may have predictive validity on disease complication. As the study design was cross-sectional, the causal relationship between TCM pattern and diabetic complication should be further examined in longitudinal studies.

The paper “Cerebral Activity Changes in Different Traditional Chinese Medicine Patterns of Psychogenic Erectile Dysfunction Patients” showed that, compared to patients with* liver*-*qi stagnation* and* spleen deficiency*, patients with* kidney*-*yang deficiency* showed an increased activity in bilateral brainstem, cerebellum, hippocampus, and the right insula, thalamus, and middle cingulate cortex and a decreased activity in bilateral putamen, medial frontal gyrus, temporal pole, and the right caudate nucleus, orbitofrontal cortex, anterior cingulate cortex, and posterior cingulate cortex. As the main difference in cerebral activity between the TCM patterns is in the brain regions that are responsible for emotional modulation, the authors believed that TCM classification might help disease subclassification, which could be used to provide personalized treatment.

In the paper “Analysis and Recognition of Traditional Chinese Medicine Pulse Based on the Hilbert-Huang Transform and Random Forest in Patients with Coronary Heart Disease,” the authors introduced an objective pulse measurement, which could distinguish patients with coronary heart disease and healthy controls. Further studies are needed to test the reliability and validity of the pulse characteristics. Another paper titled “Correlations between Phlegm Syndrome of Chinese Medicine and Coronary Angiography: A Systematic Review and Meta-Analysis” showed that phlegm syndrome can help to determine the severity of coronary heart disease.

Lastly, in the paper “Prescription of Chinese Herbal Medicine in Pattern-Based Traditional Chinese Medicine Treatment for Depression: A Systematic Review,” the authors found that* liver qi depression*,* liver depression and spleen deficiency*,* dual deficiency of the heart and spleen*, and* liver depression and qi stagnation* were the most common TCM patterns in people with depression. The authors identified several pattern-based TCM treatments that could be further examined. Xiaoyao decoction was the most frequently used herbal formula for the treatment of* liver qi depression* and* liver depression with spleen deficiency*, while Chaihu Shugan decoction was often used for* liver depression and qi stagnation*. The authors called for more high quality studies incorporating TCM pattern differentiation and treatment principle to examine the efficacy of TCM treatments and the additional benefit of pattern differentiation. Future studies incorporating expertise in various disciplines are needed to further examine the evidence base of TCM classification in patient care.

